# Effects of Chronic REM Sleep Restriction on D_**1**_ Receptor and Related Signal Pathways in Rat Prefrontal Cortex

**DOI:** 10.1155/2015/978236

**Published:** 2015-02-22

**Authors:** Yan Han, Xiaosa Wen, Fei Rong, Xinmin Chen, Ruying Ouyang, Shuai Wu, Hua Nian, Wenling Ma

**Affiliations:** ^1^Department of Neurology, Changhai Hospital, Second Military Medical University, Shanghai 200433, China; ^2^Department of Environmental Hygiene, Second Military Medical University, Shanghai 200433, China; ^3^Minhang District Center for Disease Control and Prevention, Shanghai 201101, China; ^4^Shanghai Sanatorium for Naval Retired Cadres, Shanghai 200434, China; ^5^Pharmaceutical Center of Yueyang Hospital, Shanghai University of Traditional Chinese Medicine, Shanghai 200433, China

## Abstract

The prefrontal cortex (PFC) mediates cognitive function that is sensitive to disruption by sleep loss, and molecular mechanisms regulating neural dysfunction induced by chronic sleep restriction (CSR), particularly in the PFC, have yet to be completely understood. The aim of the present study was to investigate the effect of chronic REM sleep restriction (REM-CSR) on the D_1_ receptor (D_1_R) and key molecules in D_1_R' signal pathways in PFC. We employed the modified multiple platform method to create the REM-CSR rat model. The ultrastructure of PFC was observed by electron microscopy. HPLC was performed to measure the DA level in PFC. The expressions of genes and proteins of related molecules were assayed by real-time PCR and Western blot, respectively. The general state and morphology of PFC in rats were changed by CSR, and DA level and the expression of D_1_R in PFC were markedly decreased (*P* < 0.01, *P* < 0.05); the expression of phosphor-PKAc*α* was significantly lowered in CSR rats (*P* < 0.05). The present results suggested that the alteration of neuropathology and D_1_R expression in PFC may be associated with CSR induced cognitive dysfunction, and the PKA pathway of D_1_R may play an important role in the impairment of advanced neural function.

## 1. Introduction

As one of the most striking problems in modern society, sleep disorder greatly shortens the time of sleep [1]. Sleep disorder can lead to sleep restriction/sleep deprivation, further resulting in hazardous physiological effects [2]. Sleep deprivation is particularly serious among people who are in some special professions, such as air transport, seafaring, emergency medicine, military contingency, and social security. Sleep deprivation can reduce job performance and cause cognitive impairments in humans including deficits in attention, learning, and memory, executive function, and alertness as well as emotional reactivity [3]. Compared with acute sleep restriction (ASR), chronic sleep restriction (CSR) which is induced by long-term intermittent sleep deprivation or sleep fragmentation is more prevalent and can lead to more serious consequences [4]. However, the exact neurochemical mechanism underlying CSR-induced advanced neuronal dysfunction has not been fully understood.

The prefrontal cortex (PFC) plays an important role in many higher-order executive tasks. It is involved in learning [5], memory [6], cognitive flexibility [7], and emotional processing. The PFC mediates cognitive responses that are sensitive to disruption by sleep loss [8]; therefore, PFC-mediated cognitive abilities might be impaired by sleep restriction. Previous studies have found reduced cognitive function with reduced neuronal activation following sleep loss in the PFC [9] and indicated that sleep loss induces alteration in synaptic plasticity and membrane excitability in the cortex [10], which might be modulated by dopaminergic system. PFC receives dopaminergic projections from both the substantia nigra and the ventral tegmental area [11]. Dopaminergic innervation constitutes a major regulating system that modulates higher cognitive function. In addition to modulating neural activity within PFC, dopamine (DA) also alters activity-dependent neural plasticity; these changes might play a pivotal role in learning, memory, and cognitive function [12, 13]. Therefore, DA and its receptors might be associated with the changes of synaptic excitability and plasticity in PFC during sleep restriction.

The diverse physiological actions of DA are mediated by five distinct subtypes, which are commonly divided into two classes: the D_1_-like receptors (D_1_Rs, comprised of D_1_ and D_5_ receptors) and the D_2_-like receptors (D_2_, D_3_, and D_4_ receptors) [14]. Within cortex, the D_1_Rs are expressed in prefrontal V-VI layer pyramidal neurons and I–III layer interneurons. The expression of D_1_Rs is enriched in the PFC of both primates and rodents, suggesting an important role in specifically circuit functions in PFC. D_1_ receptor (D_1_R) is a principal member of D_1_Rs, and, in PFC, D_1_R has been shown to modulate many neural activities [15, 16]. As a G-protein-couple receptor, D_1_R can be coupled to the G protein Gs and activate adenylyl cyclase and regulate the expression of cAMP response element binding (CREB) protein which is a nuclear transcription factor, modulating the functions of neurons. This regulation can be performed through several pathways such as phosphate kinase A (PKA) and mitogen activated protein kinase (MAPK) pathways [17]. However, less is known about how CSR induces the changes of neuronal ultrastructure in PFC and which pathway of D_1_R plays the major role in PFC during the cognitive dysfunction. In this study, we employed transmission electron microscope to observe the ultrastructure of PFC and used PCR and Western blot to measure the expression of related signal molecules. The design of the study aimed at exploring preliminarily the modulatory mechanism of cognition underlying D_1_R and its signal pathways in PFC during CSR.

## 2. Materials and Methods

### 2.1. Animals and Groups

Sixty male Sprague-Dawley rats, weighing 300 ± 20 g, were purchased from the Animal Center of Second Military Medical University (SMMU), Shanghai, China. All treatments and experimental procedures were approved by the Animal Ethics Committee of SMMU in accordance with the international guidelines for animal experiments. After a week of adaptive feeding and 3 days of training, 16 rats were rejected due to escape latency exceeding 90 seconds in each trial in water maze or failure to stand on the platform constantly in the sleep deprivation tank. The screening regimen and requirements were described in the previous study [18]. The remaining 44 animals were randomly assigned to the two groups: the treatment control (TC) group and the chronic sleep restriction (CSR) group, each consisting of 22 animals.

### 2.2. Experimental Procedures

The animals were housed in a room (22 ± 1°C; 12 h light-dark cycle, light on at 8:00 a.m., light off at 8:00 p.m.) with normal feed and water. The CSR model was created by modified multiple platform method as stated earlier [19]. Eight narrow platforms (diameter 6.5 cm) or a grid floor was placed inside a water tank. Six rats in the CSR group were forced to stand on the platforms surrounded by water for 18 h (beginning at 16:00) daily; at least two platforms in each tank are empty, thus allowing animals to move around. Animals in the TC group were placed on the grid, thus allowing them to sleep and move freely in a similar water environment. This process continued for 21 days to create the chronic REM sleep restriction (REM-CSR) model. Tufik and his colleagues employed the method of EEG monitoring to discover that this sleep restriction led to a complete suppression of REM sleep during the 18 h sleep deprivation period each day, which persisted throughout the whole restriction period [1, 20]. Food and water were available through a grid placed on top of the water cage during the time of sleep restriction.

### 2.3. Preparation of PFC

**Figure 1:**
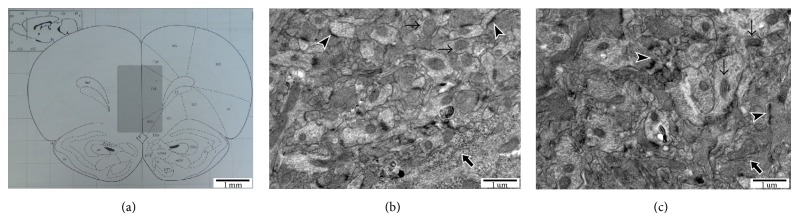
Transmission electron microscopic results of PFC (*n* = 4). (a) The shadow area in the middle of cross-section is the objective PFC observed by electron microscope; compared to the TC group (b), the CSR group (c) showed that the number of mitochondria (thin arrows) was decreased and the structure of mitochondrial cristae was fuzzy and ruptured; the postsynaptic density (arrowheads) became much thinner or disappeared; the rough endoplasmic reticulum (thick arrows) structure(s) became fuzzy.

After 21 days of CSR, the animals were perfused with 4°C saline through the ascending aorta under chloral hydrate anesthesia (400 mg/Kg, i.p.). PFC from 18 animals in each group (including prelimbic medial cortex and infralimbic medial cortex) was dissected under microscope (Figure 1(a)). The samples were immediately frozen in liquid nitrogen and stored at −80°C for subsequent experiment. Meanwhile, samples of the last 4 rats in each group were fixed with 2.5% glutaraldehyde and 4% paraformaldehyde for the observation of ultrastructure.

### 2.4. Observation of Electron Microscope

The isolated PFC tissues were fixed with 1% osmium tetroxide and then dehydrated and embedded in resin. The 80 nm sections were cut and double stained with uranyl acetate and lead citrate. The ultrastructure of PFC was observed under transmission electron microscope (H-7650, Hitachi, Japan) and images were recorded.

### 2.5. The DA Concentration in PFC

The DA concentration was detected by High-Performance Liquid Chromatography with electrochemical detection (ESA, USA, *n* = 8 for each group). The PFC tissues were sonicated in 0.5 mL of 0.1 M perchloric acid and DA extraction was performed. The supernatant was filtered through a 0.22 Millex-GV filter and transferred to an autosampler tube prior to injection. Detection was performed with an ESA Coulochem III electrochemical detector (ESA, USA). The area under the curve (AUC) of standard solutions was used for the comparison of dopamine concentrations in the samples.

### 2.6. RNA Isolation and Quantitative Real-Time PCR

The mRNA for 7 signal transduction molecules was examined by real-time PCR (*n* = 6 for each group). Total RNA was extracted from PFC tissue with Trizol reagent (Invitrogen), according to the instructions of the manufacturer. RNA yield and quality were determined spectrophotometrically by A260/A280 ratio. After reverse transcription, the real-time PCRs were done in a 20 uL reaction mixture system. Amplification was performed by the Applied Biosystems 7500 Real-Time PCR System (ABI, USA) with an initial denaturation of 5 min at 95°C, followed by 45 cycles of 95°C for 15 s, 60°C for 30 s, and 72°C for 30 s. At the end of the amplification phase, a melting curve analysis was performed to ensure the amplification of a single PCR product. Primers used in the amplification were designed and synthesized by Sangon Biotech (Shanghai) Co., Ltd. The sequences of 7 gene primers were shown in Table 1.

### 2.7. Protein Extraction and Western Blotting

The frozen PFC samples were dropped into SDS extraction buffer and homogenized with an ultrasonic cell disrupter and then boiled and centrifuged. The supernatant was transferred to a new tube, and, after determination of the protein concentrations, the samples were then diluted to SDS extraction buffer and stored at −20°C for later experiments. Western blotting (*n* = 4 for each group) was used to measure the protein expressions of D_1_R (1 : 500, Sigma, D2994), phosphorylated PKAc*α* (1 : 1000, CST, #5661), PKAc*α* (1 : 1000, abcam, ab76238), phosphorylated ERK1/2 (1 : 1000, Bioworld, BS5016), ERK1/2 (1 : 1000, Bioworld, BS1112), phosphorylated CREB (1 : 1000, CST, #9198), CREB (1 : 1000, CST, #9197), and *β*-actin (1 : 1000, Santa Cruz, sc-1616r). The sample proteins were separated by 10% SDS-polyacrylamide gel electrophoresis and transferred to nitrocellulose membrane (Millipore, USA). The membranes were blocked with 5% nonfat dry milk in Tris buffered saline (TBS) for 2 h at room temperature and incubated with the primary antibodies in TBS with 5% dry milk overnight at 4°C. The membranes were washed with TBS plus 0.1% Tween-20 (TBS-T) three times (10 min each) and incubated for 2 h at room temperature with different horseradish peroxidase-conjugated secondary IgG antibodies (1 : 3000, KPL, anti-rabbit or anti-mouse) and washed again in TBS-T (10 min ×3). Blots were revealed by ECL advanced kit (Thermo Fisher, USA). To normalize protein content, blots were probed with corresponding *β*-actin, and the ratio to *β*-actin was calculated.

### 2.8. Statistical Analysis

All the assessment data were analyzed by a two-tailed Student's *t*-test for the comparison of mean values between the two groups, using GraphPad Prism 5.01 (GraphPad Software, Inc., USA). Values were expressed as mean ± standard deviation and differences were considered to be significant when *P* < 0.05.

## 3. Results

### 3.1. The Change of Behavioral Ability and General State of CSR Rats

In this study, we found that the learning and memory ability, stamina, and locomotor activity in rats were significantly reduced in the CSR group, as compared with the TC group. This result repeats our previous reports [18]. In addition, during the course of sleep restriction, we also found that CSR rats displayed increased excitability and augmented alertness to environmental stimulation in the early phase (CSR 1st−7th d), and the animals showed irritability and bite and attack enhancements in the middle stage (CSR 8th−14th d). In the late phase (CSR 15th−22th d), the rats demonstrated emaciation, response slowness, arched back, and trembling and fell into water more frequently.

### 3.2. The Pathological Changes and DA Levels in PFC

**Figure 2:**
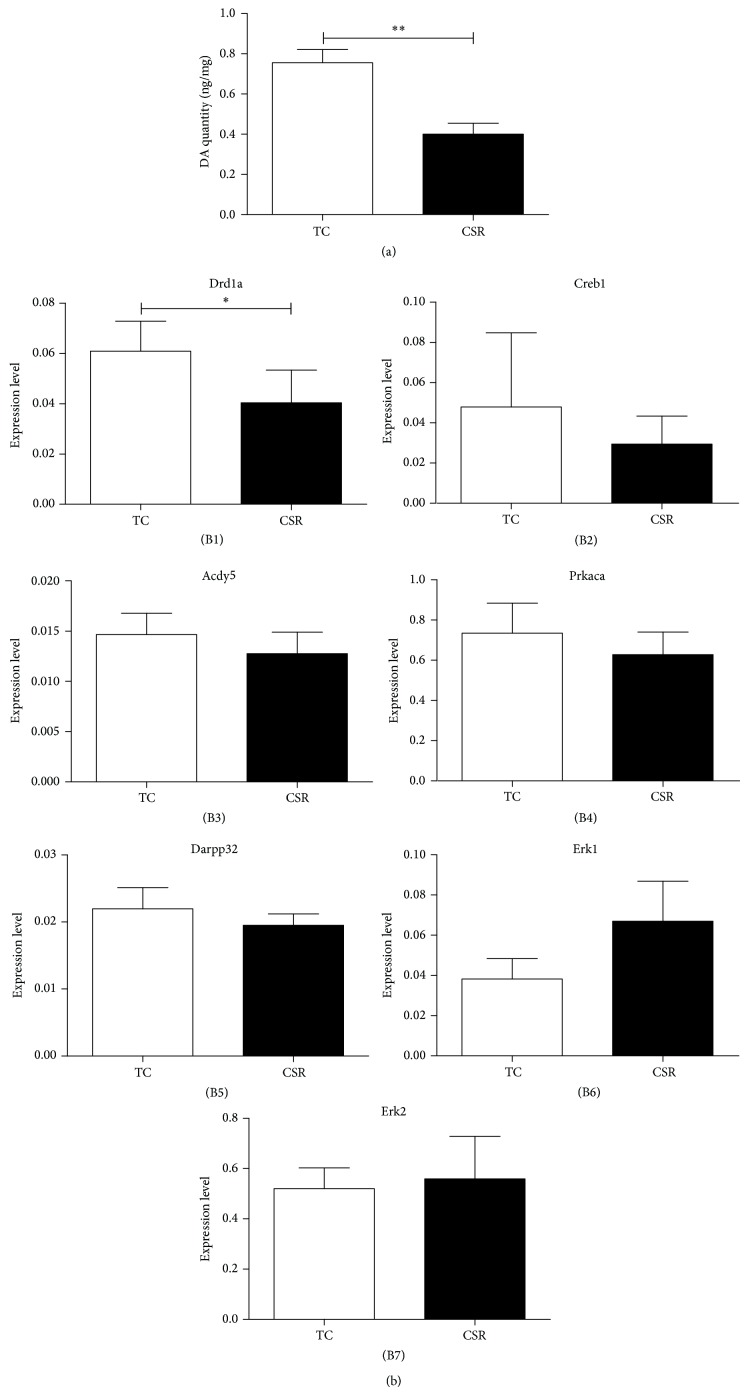
(a) The results of DA content in the two groups (*n* = 8). Compared to that of the TC group, the DA content in the CSR group was notably reduced. (b) Effects of CSR on the expressions of some genes in PFC (*n* = 6). CSR notably reduced the Drd1a in the CSR rats (B1). The expressions of Creb1, Acdy5, Prkaca, Darpp32, Erk1, and Erk2 in PFC showed no significant differences between the CSR and TC groups (B2–B7). TC: treatment control; CSR: chronic sleep restriction. ^∗^ *P* < 0.05, and ^∗∗^ *P* < 0.01.

To assess the morphological changes, the ultrastructures of PFC in the two groups were observed under the transmission electron microscope according to the previous method [21]. We randomly examined 5 fields in each group. The mitochondrial impairment was identified by observing the vague or disappeared cristae and the fragmentation of membrane. We found that only 18.3% (11/60) of the mitochondria in the TC group was damaged, while the damage rate in the CSR group was 51.0% (26/51). The damages of postsynaptic density were identified when the structure of synapse became much thinner or disappeared. Both the number and the rate of damaged synapses in the CSR group (44.4%, 12/27) were obviously increased, compared with those of the control group (24.1%, 7/29). Moreover, the structure of rough endoplasmic reticulum became fuzzy (Figure 1). Meanwhile, when compared with that of the TC group, the DA concentration of PFC in the CSR group was notably decreased (*P* < 0.01, Figure 2(a)).

### 3.3. Effects of CSR on the Expression of Key Molecules mRNAs in D_1_R Pathway

In addition to D_1_R, we also chose 6 other key molecules in the D_1_R signal pathways and detected their mRNA expression. Drd1a was the gene of D_1_R; Acdy5, Prkaca, and Darpp32 are the crucial regulators of PKA pathway; Erk1 and Erk2 play an essential role in the MAPK pathway; Creb1 is a transcription factor and is involved in the regulation of learning and memory. The results showed that only Drd1a was significantly reduced by CSR (*P* < 0.05, Figure 2(B1)), and the expressions of other genes showed no differences between the two groups during CSR (Figure 2(B2)–(B7)).

### 3.4. Effects of CSR on the Expression of Key Molecules Proteins in D_1_R Pathway

**Figure 3:**
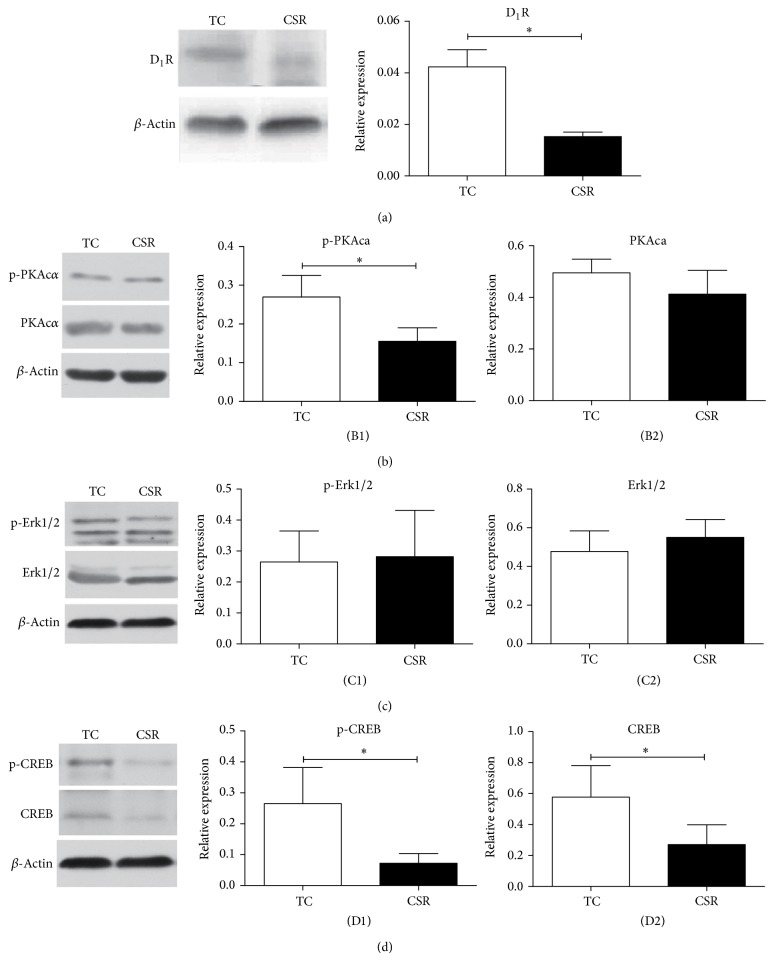
Effects of CSR on the expressions of some proteins in PFC. The D_1_R (a) and p-PKAca (B1) in the CSR rats were significantly decreased. CSR did not influence PKAca (B2), P-Erk1/2 (C1), and Erk1/2 (C2) in PFC. Both the p-CREB (D1) and CREB (D2) in PFC were notably reduced by CSR. ^∗^ *P* < 0.05, *n* = 4 per group.

The D_1_R protein expression in the CSR group was significantly decreased, as compared to that in the TC group (*P* < 0.05, Figure 3(a)). The phosphor-PKAca was also notably reduced in the CSR rats, as compared to the corresponding control (*P* < 0.05, Figure 3(B1)). Both the phosphor-CREB and CREB in the CSR group were obviously decreased as compared with CREB in the TC group (*P* < 0.05, Figure 3(D1)/(D2)). There were no significant changes in PKAca (Figure 3(B2)), phosphor-Erk1/2 (Figure 3(C1)), or total Erk1/2 (Figure 3(C2)) between the CSR and the control group.

## 4. Discussion

According to the results of our preliminary experiment, there was only one control group in the study. The blank control (cage control) was rejected due to its similar outcome to the treatment control. The modified multiple platform method used to create the CSR model had been repeatedly tested by previous researches [1]. This method had been further modified in an attempt to eliminate the social isolation and the restriction of movement. The treatment control group for the deprivation environment consisted of animals lying on a grid placed inside the water tank, which is commonly used as a control for the confounding factors inherent to this technique [19].

Sleep deprivation has a broad variety of effects on performance and neural function that manifest themselves at different levels of description [22]. Our previous study demonstrated that REM-CSR can significantly drop the learning and memory ability, stamina, and locomotor activity. And the results also showed that, after one week of CSR, the ability of learning and memory began to decline and gradually reduced in the second and third weeks [18]. Those results are consistent with many other studies [23, 24]. This research also observed that the general state and mental condition of rats were affected by REM-CSR and changed with the prolonged restriction.

Our latest study pointed out that the cognitive dysfunction of the CSR rat was related to the pathological change of ultrastructure of hippocampus [18]. This research is the first to observe the PFC neuronal degeneration after REM-CSR, and we also found that the mitochondria and endoplasmic reticulum in the PFC were destructed. The rate of mitochondrial damage caused by REM-CSR in PFC was 51%, which was much more than those of hippocampus. Furthermore, the same destruction of endoplasmic reticulum and postsynaptic density in PFC demonstrated that REM-CSR can cause similar damage of PFC and hippocampus. Different exposure or stress can all lead to the destruction of mitochondria, such as swelling and the disappearance or fuzziness of crista. And the damage of mitochondria was associated with the degeneration, apoptosis, and cell death of neurons, which can reduce the discharge activity of neurons. The results indicated that, through altering the structure of neurons, REM-CSR might reduce the neuronal excitability and then affect the function of PFC. PFC is involved in working memory, decision making, and vigilance maintenance [25, 26]. It could be therefore inferred that PFC structural destruction might play a crucial role in the CSR-induced advanced neuronal dysfunction.

The neuronal degeneration of PFC was associated with the reduction of neuronal activity [8, 27]. Moreover, previous studies have indicated that the activation of D_1_R increases neuronal activity, suggesting that the D_1_R may be involved in the modulation of neuronal activity [28]. The present study displayed that DA concentration and the expression of D_1_R mRNA and protein were significantly reduced after REM-CSR, suggesting that the CSR-induced changes of PFC structure and activity may be associated with the alteration of D_1_R expression and its signal pathways. Therefore, the DA and D_1_R in the PFC might play an important role in the CSR-induced advanced neural dysfunction, and elevation of DA concentration or stimulation of D_1_R in the PFC might improve the cognitive dysfunction during the CSR process.

**Figure 4:**
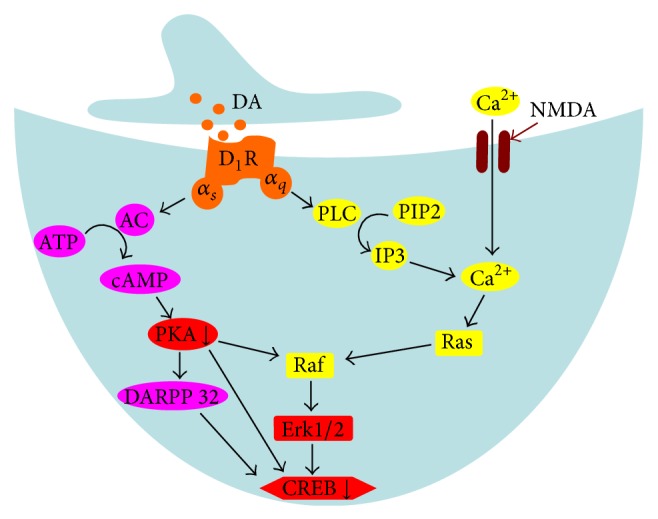
The scheme of signal pathways of D_1_R. This simplified view of the signal pathways shows major downstream mediators between the two major pathways of D_1_R. D_1_R regulates CREB through PKA pathway (expressed by purple molecules) and MAPK pathway (yellow members), and the key molecules are marked by red. CREB was significantly decreased by CSR, while PKA showed a notable reduction (expressed by downward arrow).

D_1_R regulates the nuclear transcription factor via two major pathways: PKA and MAPK pathways, and PKA and extracellular signal-regulated kinase (ERK1/2) are the key regulatory molecules in PKA and MAPK pathways, respectively (Figure 4). Studies have shown that the ability of D_1_R to increase both the persistence and early magnitude of long-term potentiation (LTP) was associated with activation of PKA pathway [29], and other researchers found that the decreased phosphorylation of DARPP-32 and CREB, which were also the key signal molecules in PKA pathway, was linked to the notable impairment of delayed spatial memory retrieval caused by selective D_1_R antagonist in the prefrontal cortex [28]. In many cases, ERK1/2 was involved in memory consolidation. Guan et al. [30] indicated that decreased ERK1/2 activation in the hippocampus was associated with sleep deprivation-induced spatial memory impairment, but the alterations of ERK1/2 were not observed in the cortex. In this study, we demonstrated that REM-CSR decreased the phosphorylation of PKA and CREB but did not alter the level of phosphor-ERK1/2 (Figure 4), suggesting that the reduction of DA concentration and D_1_R expression induced by REM-CSR might impair the function of PFC by repressing PKA pathway activity but not the MAPK pathway. These results provide new insights in the molecular mechanisms involved in D_1_R-dependent function modulation of PFC, where PKA pathway plays a pivotal role in the synthesis of CREB during REM-CSR.

However, Chen et al. [27] once discovered that the form of D_1/5_ receptor-mediated LTP of the intrinsic excitability relied on activation of Ca^2+^-dependent intracellular signal in PFC, indicating that the D_1/5_ receptor-mediated phosphoinositol (PI) pathway, including the activation of PLC, intracellular Ca^2+^ elevation, and the activation of Ca^2+^-dependent CaMKs and PKC, might be associated with the regulation of overall excitability of PFC neurons. In our future studies, we plan to further demonstrate the regulatory mechanism of PI pathway in PFC dysfunction induced by CSR.

## 5. Conclusions

This study demonstrated that PFC is associated with the changes in behavioral performance and the impairments of cognitive function induced by REM-CSR, and the DA and its D_1_R in PFC might be involved in the regulation of this process. A key finding in this research is the reduction of the level of PKA and CREB protein phosphorylation, which indicates that PKA pathway in PFC seems to play a major role in the cognitive impairment of REM-CSR. However, mechanism for the modulation of PFC under CSR needs to be further clarified in the future.

## Figures and Tables

**Table 1:** The sequences of 7 primers in real-time PCR.

Primer name		Sequence (5′ to 3′)
Drd1a	Forward	GCGAGCACCACAAAGCC
	Reverse	GGAAACTAGATTCAAACCCACAGA

Creb1	Forward	TTGTTGTTCAAGCTGCCTCTG
	Reverse	TGCTGTGCGAATCTGGTATGT

Adcy5	Forward	CAACTACCTGAACGGGGACTAT
	Reverse	CGATGCTGTGCTCCTTGA

Prkaca	Forward	CCCACTCCCTAAATCCATTCTG
	Reverse	AAGCCCAGTTCCTTCCTTGAC

Darpp32	Forward	CTCCCCAGAAACCCACTCT
	Reverse	GCAAACACAGACCAGCCTTAGT

Erk1	Forward	CTGGAATGGAAGGGCTATGAC
	Reverse	CAACAGGATGAGTAGGGCAGAG

Erk2	Forward	GCGTTCAGATGTCGGTGTC
	Reverse	CAAAGGAGTCAAGAGTGGGTAAG

Rat-gapdh	Forward	CAGTGCCAGCCTCGTCAT
	Reverse	AGGGGCATCCACAGTCTTC
